# Efficacy of virtual reality exposure therapy and eye movement desensitization and reprocessing therapy on symptoms of acrophobia and anxiety sensitivity in adolescent girls: A randomized controlled trial

**DOI:** 10.3389/fpsyg.2022.919148

**Published:** 2022-09-15

**Authors:** Parisa Azimisefat, Ad de Jongh, Soran Rajabi, Philipp Kanske, Fatemeh Jamshidi

**Affiliations:** ^1^Department of Psychology, Persian Gulf University, Bushehr, Iran; ^2^Academic Centre for Dentistry Amsterdam (ACTA), University of Amsterdam and VU University Amsterdam, Amsterdam, Netherlands; ^3^Clinical Psychology and Behavioural Neuroscience, Faculty of Psychology, Technische Universität Dresden, Dresden, Germany

**Keywords:** VRET, EMDR therapy, anxiety sensitivity, acrophobia, specific phobia, anxiety disorders

## Abstract

**Background:**

Acrophobia is a specific phobia characterized by a severe fear of heights. The purpose of the present study was to investigate the efficacy of two therapies that may ameliorate symptoms of acrophobia and anxiety sensitivity, i.e., virtual reality exposure therapy (VRET) and eye movement desensitization and reprocessing (EMDR) therapy with a Waiting List Control Condition (WLCC).

**Methods:**

We applied a three-armed randomized controlled pre-post-test design with 45 female adolescent students. Students who met DSM-5 criteria for acrophobia were randomly assigned to either VRET (N = 15; *M*age = 17.26; SD = 1.32), EMDR (N = 15; *M*age = 17.15; SD = 1.57), or a WLCC (N = 15; *M*age = 17.50; SD = 1.26). The study groups were evaluated one week before the intervention and one week after the last intervention session regarding symptoms of acrophobia (Severity Measure for Acrophobia) and anxiety sensitivity (Anxiety Sensitivity Index).

**Results:**

The data showed that both the application of VRET and EMDR therapy were associated with significantly reduced symptoms of acrophobia (d = 1.03 for VRET and d = 1.08 for EMDR) and anxiety sensitivity (d = 1.15 for VRET and d = 1.13 for EMDR) in comparison to the Waiting List.

**Limitations:**

The sample consisted only of adolescent women. Due to the recognizable differences between the two interventions, the therapists and the participants were not blind to the conditions.

**Conclusion:**

The results suggest that both VRET and EMDR are interventions that can significantly improve symptoms of acrophobia and anxiety sensitivity in female adolescents.

**Clinical Trial Registration:**

https://www.irct.ir/trial/57391, identifier: IRCT20210213050343N1.

## Introduction

Anxiety disorders are among the most prevalent mental health conditions, and specific phobia is one of the most prevalent anxiety disorder (Eaton et al., 2018). The lifetime prevalence of specific phobias has been estimated to be between three and 15 % (Eaton et al., 2018), with fear of snakes (35%) and heights (31%) being the most common subtype (Oosterink et al., 2009).

Severe fear of heights, or acrophobia, is a phobia subtype that involves a psychological barrier to confrontations with heights (Suyanto et al., 2017) often leading to the avoidance of height-related objects and situations (i.e., stairs, terraces, etc.) (Davey, 1997). Due to the persistent avoidance of an extensive range of places that are commonly encountered in daily living, acrophobia negatively impacts social life as well (Watson, 1999). In addition to these negative social effects, acrophobia has high comorbidity rates with a wide variety of mental health conditions, such as major depression, anxiety disorders, and specific fears including fear of elevators and fear of flying (Clark et al., 1992; Jacob et al., 1997; Curtis et al., 1998; Muris et al., 1999; Choy et al., 2007).

An underlying physiologic abnormality may contribute to problems with balance control, height vertigo, visual dependence, and space and motion discomfort in individuals suffering from acrophobia (Brandt et al., 1980; Jacob et al., 1993, 1995, 1997). According to (Davey et al. 1997), acrophobia may develop in a manner similar to panic disorder (Davey, 1997). Acrophobia has been associated with increased sensitivity to physiological symptoms, including dizziness, feeling short of breath, and heart palpitations (Davey et al., 1997). Such cognitive biases may lead individuals with acrophobia to interpret bodily sensations to movement in height-related situations as threatening (Taylor, 2019). These interpretations might cause individuals to associate these signals with a higher likelihood of a forthcoming catastrophic fall, thereby enhancing and maintaining the fear response (Davey et al., 1997).

Although the role of anxiety sensitivity in acrophobia is not well studied individuals with fear of heights suffer from a natural disturbance of a physiologic response (e.g., balance control) when confronted with heights (Boffino et al., 2009). Because bodily sensations associated with a loss of balance can easily be mistaken as harmful experiences, causing anxiety or fear to intensify, anxiety sensitivity may play a role in the acquisition and maintenance of acrophobia.

Cognitive-behavioral therapy is the first line of treatment for fears and phobias (Gromer et al., 2018). This treatment approach is based upon the principle that when people are frequently exposed to stimuli that provoke anxiety (thoughts, objects, or situations) with no subsequent negative consequences, individuals show a reduction of anxiety symptoms over time (Herrmann et al., 2017; Baker et al., 2020; Pittig et al., 2021). Although exposure therapy is widely used, it can be costly and time-consuming for therapists who wish to incorporate phobic stimuli in their treatment (Freeman et al., 2018; Heinig et al., 2021). More recently, Virtual Reality Exposure Therapy (VRET) has been introduced and developed as an emerging technology that is increasingly being used to treat patients with specific phobias (Carl et al., 2019), especially in patients with acrophobia (Ling et al., 2014). During VRET sessions, patients are assisted in frequent confrontations with stimuli and situations that trigger a fear response in a virtual and controlled environment until their fear subsides (Krijn et al., 2007). Because a meta-analysis showed that VRET is an effective therapy for acrophobia (Carl et al., 2019), we opted a waiting list as the control condition to explore the efficacy of VRET in our study.

Another evidence-based therapeutic intervention, eye movement desensitization and reprocessing (EMDR) therapy (de Jongh et al., 2019), has been found useful in the treatment of post-traumatic stress disorder (PTSD), for which it had originally been developed, and also in the treatment of specific phobias (i.e., flight anxiety or dental phobia) (Doering et al., 2013; Triscari et al., 2015). There are convincing reasons to apply EMDR therapy in the treatment of specific phobias. For instance, individuals with specific phobias demonstrate commonalities with PTSD such as experiencing intrusive, disturbing, and frightening memories of earlier events associated with their phobic condition (De Jongh et al., 2002; Oosterink et al., 2009). As EMDR therapy has been found to alleviate the disturbance of such memories with a lasting effect on the phobic symptoms (De Jongh et al., 2002; De Roos and De Jongh, 2008; Doering et al., 2013; Lapsekili and Yelboga, 2014; Faretta and Dal Farra, 2019; Meentken et al., 2020), it may also be a candidate for the treatment of acrophobia. However, it is important to note that Cuijpers et al. (2019), in their meta-analysis, concluded that, based on the current empirical basis, therapists need to exercise caution in applying EMDR therapy to various mental health conditions other than PTSD (Cuijpers et al., 2020). The present study aims to contribute to the literature and bridge this important gap.

The purpose of the present study was to investigate the efficacy of virtual reality exposure therapy (VRET) and eye movement desensitization and reprocessing (EMDR) therapy on symptoms of acrophobia and anxiety sensitivity. The study design of this randomized controlled study included a screening phase, a pre-treatment assessment phase, a randomization phase, an intervention phase consisting of six weekly treatment sessions, and a post-treatment assessment (one week after the last treatment session). It was hypothesized that both VRET and EMDR therapy would be associated with statistically significant reductions in symptoms of both acrophobia and anxiety sensitivity, and significantly more symptom decline in comparison to the waiting list control condition. Due to insufficient statistical power to detect small effects, we could not formulate an *a priori* hypothesis regarding the differential efficacy of VRET and EMDR therapy.

## Materials and methods

### Study design

**Figure 1:**
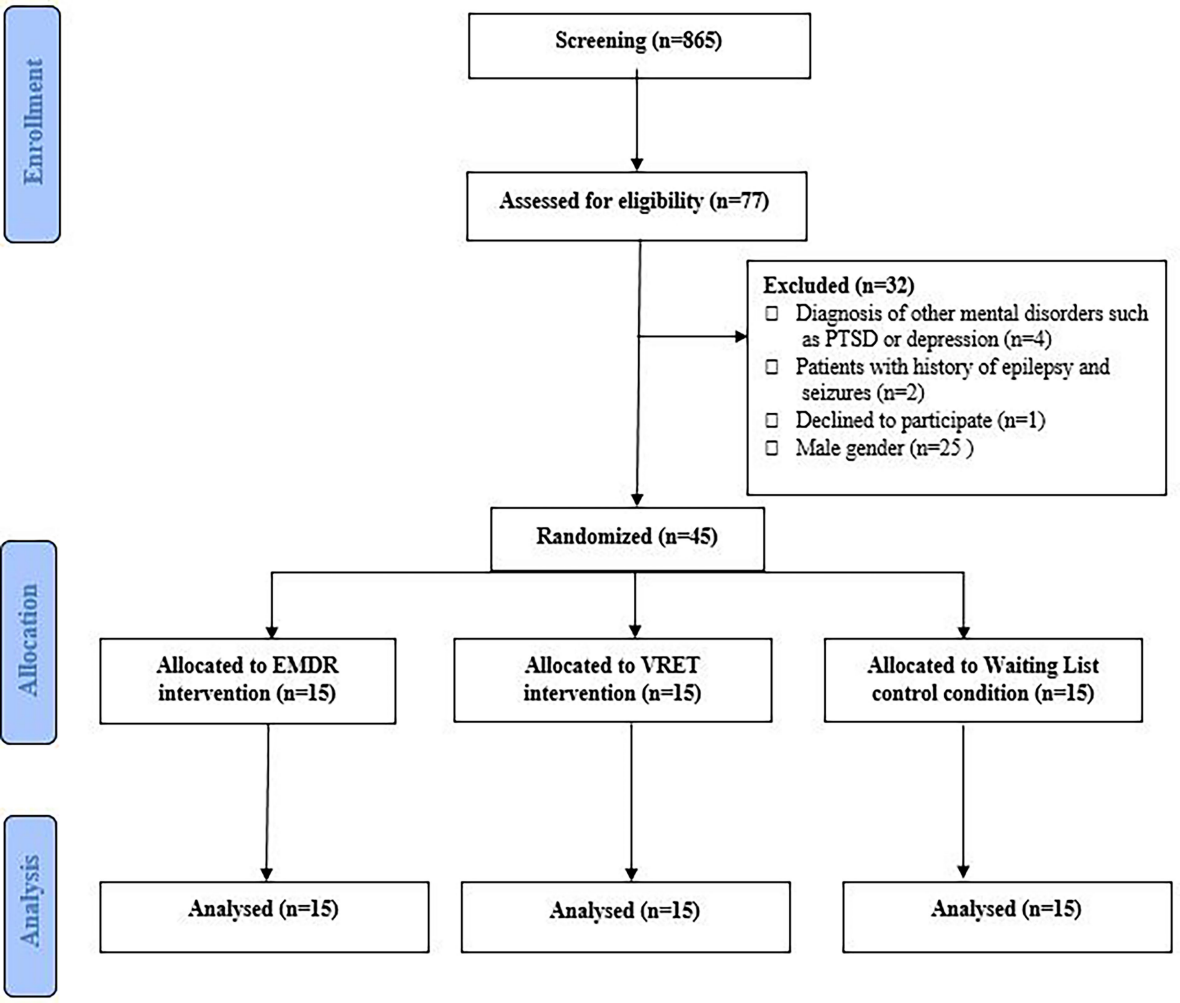
CONSORT flowchart for study design.

We conducted a randomized controlled trial (RCT) including 45 female adolescents in the department of Psychology at the Persian Gulf University (PGU). The study involving human participants was reviewed and approved by the Research Ethic Committees of Bushehr Province University of Medical Science (Reference: IR.BPUMS.REC.1400.036). Block randomization with a block size of 15 was used for assigning the subjects into three groups: (I) VRET group, (II) EMDR group, and (III) waiting list control condition (WLCC) group. Each group consisted of 15 female adolescents who were not blind to group assignments due to the nature of the interventions. However, the data collector was blind to group assignments. Both VRET and EMDR therapy were carried out by P. Azimisefat who is a CBT and EMDR therapist with a master’s degrees in psychology. She was supervised in both treatments by Dr. S. Rajabi. The interventions are described using the TIDierR checklist (Hoffmann et al., 2014; see Appendix 2). Figure 1 shows a CONSORT flowchart for the study design. Multimedia Appendix 1 contains the SPIRIT (Standard Protocol Items: Recommendations for Interventional Trials) flow diagram.

### Patients

#### Inclusion criteria

Participants were eligible for inclusion in the study if they had a SMA score greater than or equal to 50 (n = 77), met all diagnostic criteria of having a specific phobia (i.e., acrophobia) based on the Structured Clinical Interview for DSM-5 (SCID-5) (First, 2015), and provided written informed consent which was completed independently or by their parents. The cut-off of 50 was chosen based upon the lifetime prevalence of acrophobia, estimated as 6.0% (Kapfhammer et al., 2015), and the 94-percentile value.

#### Exclusion criteria

Exclusion criteria included: the male gender, patients with hearing or visual impairment such as stereoscopy blindness or nystagmus, presence of any other mental disorders as assessed using the SCID-5, (First, 2015), presence of specific physiological illnesses such as heart, lung and respiratory diseases, epilepsy and seizures, developmental or intellectual disability, cognitive impairment, known balance disorders such as vertigo, addiction to alcohol or drugs, or other current psychological treatment.

### Procedure

Following baseline screening by multistage cluster, of 865 male and female adolescents (16–18 years old, Mage = 17.10; SD = 1.25), 77 (8,90%) scored above the cut-off score of the SAM and were invited to complete the SCID-5 (First, 2015). During the clinical interview, we asked questions to all participants about their fear, anxiety, or avoidance, which typically lasted for six months or more. All participants experienced acrophobic worries, and we asked them to describe the severity in three qualitative assessments: mild, average, and severe. Also, we asked them to determine how many acrophobic situations scare them. For instance, stairs, ladders, bridges, balconies, tall buildings, hills, roofs, and some recreation related to height like balloons, carousels, etc. The age, severity of acrophobia symptoms, and number of acrophobic situations in which patients experienced acrophobic symptoms in the participants who fulfilled the diagnostic criteria of specific phobia are reported in Table 1. After screening, 45 female adolescents were included in the study. Participants completed and signed the consent form after being informed about the details of the study. Parental signatures were obtained if the participants were younger than 18 years old.

**Table 1:** Demographic characteristics of the randomized controlled trial sample.

Descriptive characteristics	EMDR group	VRET group	WLCC group	F	P
Age M (SD)	16.93 (0.88)	17.20 (0.77)	16.93 (0.79)	0.528	0.593
Education attainment (N and %)					
10th Grade	6 (40.0)	3 (20.0)	5 (33.33)		
11th Grade	4 (26.66)	6 (40.0)	6 (40.0)		
12th Grade	5 (33.33)	6 (40.0)	4 (26.66)		
Severity of acrophobia symptoms (N and %)					
Mild	1 (6.66)	0 (0.00)	0 (0.00)		
Average	9 (60)	9 (60)	8 (53.33)		
Severe	5 (33.33)	6 (40)	7 (46.66)		
Number of acrophobic situations (N and %)					
Acrophobic situation #1	7 (46.66)	2 (13.33)	4 (26.66)		
Acrophobic situations #2	1 (6.66)	8 (53.33)	4 (26.66)		
Acrophobic situations #3	7 (46.66)	5 (33.33)	7 (46.66)		

M = mean, SD = standard deviation, N = number, VRET = Virtual Reality Exposure Therapy; EMDR = Eye Movement Desensitization and Reprocessing, WLCC = Waiting List Control Condition.

### Sample size

Due to the novelty of our research, comparable studies with estimated sample sizes were limited. Previous RCTs on VRET for acrophobia showed an effect size of 0.79–1.42 Cohen d (Carl et al., 2019). We conducted a sample size estimation using G-Power (Mayr et al., 2007). The minimum sample size to detect differences between matched groups with effect size (0.80), statistical power effect of 80%, α = 0.05 was n = 36 subjects. Finally, we adjusted to 15% drop-outs rates and 10% for each VR software crash during treatment to obtain a final sample size of 45 patients.

### Randomization and blinding

Participants were randomized using a random number table: VRET (n = 15), EMDR therapy (n = 15), and the WLCC (n = 15). Neither the therapist nor participants were blinded. However, the data analyst and the person conducting the assessments were blind to the participant’s group. Before the first session, the researcher collected information about the patients’ clinical history. The researcher explained the purpose of the study to the patients and made plans for future sessions. We tried to create suitable homogeneity for implementing both treatments with accurate planning. Therefore, six treatment sessions were used for both therapies and all participants in experimental conditions. Evaluations were carried out one week before and after the application of the treatment sessions to all participants who also completed questionnaires pertaining to acrophobia severity and anxiety sensitivity.

### Interventions

Following randomization, patients received either VRET or EMDR therapy approximately twice per week. Given that there were two experimental groups in this study treated with two different methods, the implementation process for each group is described separately. The WLCC group did not receive any treatment during the study, but participants in this group were introduced to the university clinic for treatment session appointments.

#### VRET intervention

VRET was conducted using a Desktop Computer with specifications; Graphics Card: ASUS NVIDIA GEFORCE GTX 1060 6GB GDDR5, CPU: Core i7-4,790 (8 M Cache, up to 4.00 GHz), RAM: 8 GB DDR4, and an Oculus Rift Dk2 Virtual Reality Headset. These tools enabled us to create a virtual environment and to provide the VR stimuli to the patient in a safe manner. We used the Unity platform for creating VR experiences and scenarios. Patients moved within the virtual environment with a VR gamepad and we used smooth locomotion to provide an immersive environment for patients. The therapist explained how to use the app and conducted therapeutic sessions by following the standard protocols of cognitive behavioral therapy for specific phobias (Scozzari and Gamberini, 2011). Four different VR scenarios were offered. Patients were confronted with one VR scenario in each session. Depending on how fast the user wanted to pass the scenarios, each session took an average of 60 min to complete. The VRET intervention in each session included two consecutive phases: a training phase and an experimental phase (see Table 2). In the final session, all VR scenarios were presented one after another. The content of cognitive-behavioral therapy was provided by the therapist using the six modules: below.

- Module 1 (Background): The therapist described what acrophobic anxiety is, provided information about the possibility of retaining safety while in high locations, educated the patient about how acrophobic anxiety develops in a VR environment, and explained how the patient can overcome it.
- Module 2 (Facing your fear): The therapist informed and educated the patient about the fear curve and how to set realistic goals to overcome his or her fear of heights.
- Module 3 (Exposure): Immersion took between twenty minutes and half an hour. To motivate the patient to engage in the VR-environment so that his or her irrational expectations could be falsified it was emphasized that VRET is in fact harmless.
- As exposure to VR scenarios may provoke anticipatory fear, the therapist explained to the patient that this is a normal response, and that exposure to a virtual environment is not in fact dangerous. Thereafter, as a manipulation check and to determine whether exposure to the anxiety provoking situations actually occurred, we measured patients’ subjective units of disturbance (SUD) levels before and after each VR scenario. The VR scenarios contained detailed 3D animations of height scenarios mentioned in Table 2. Patients could pass the VR scenario in case of a fear score below 3 based upon a 1–10 scale (with 1 representing “no or little fear” and 10 “extreme fear”). If a patient completed a VR scenario and reported a fear level of 3 or higher, the patient was requested to practice this scenario again. Each VR scenario was repeatedly presented to the patient until the patient reported that she was able to handle her anxiety or fear (i.e., a change in SUD score of ≤3 was gained), after which the next module was conducted.
- Module 4 (Catastrophic Thoughts): The therapist explained how automatic, catastrophic thoughts affect a patient’s fear and anxiety. Also, the patient was encouraged to reflect on how realistic her catastrophic thoughts were.
- Module 5 (Helping Thoughts): The therapist invited the patient to think about what unrealistic thoughts she had about the fear, explained why these thoughts were unrealistic, and helped the patient with formulating more realistic thoughts.
- Module 6 (The Next Step): The therapist encouraged the patient to apply the new knowledge so that she could start practicing exposure in the real world.

**Table 2:** The VRET protocol for specific phobias.

**Training phase**. Through the head-mounted device (HMD), the patient viewed a 3-dimensional (3D), stereoscopic, simulated high place for five minutes, in which the patient moved her head, observed the surroundings, and walked in the environment
**Experimental phase**. The standard protocols of cognitive behavioral therapy for specific phobias was conducted and the patient was exposed to four different VR scenarios (VR1 to VR4) in the following predetermined hierarchy
*(A) VR1.* The patient could walk on a high circular hill, observe the surroundings, and stand at the top of the peak to look down
*(B) VR2.* The patient was on a high roof and could walk on top of a building and go to the edge of the roof to observe the surroundings
*(C) VR3.* The patient was asked to climb a ladder and could gradually climb the ladder and look around
*(D) VR4.* The patient was riding a balloon that was gradually elevating from the ground and climbing in height. The patient could look down from the edge of the balloon. This scenario is based on a weakness in the acrophobic individual’s ability to control her stability of posture given that persons with acrophobia are afraid of the sense of movement and height at the same time
*(E) Final VRET.* In the last VR exposure, the patient was placed in a combination of the various situations to make sure that all acrophobic scenarios were performed

The purpose of selecting the VR scenarios was to gradually assist the patient in confronting a hierarchy of standard anxiety-provoking stimuli of high places.

#### EMDR intervention

EMDR therapy in the present study was based on the standard protocol for specific phobias (De Jongh, 2015),^1^ consisting of eight phase (phase 1: history taking; phase 2: preparation; phase 3: assessment; phase 4: desensitization; phase 5: installation; phase 6: body scan; phase 7: closure; and phase 8: reevaluation) performed over six one-hour sessions spread across six weeks. In the first session, we recorded information about the history of the patients’ disorder and taught the patient self-control procedures to cope with the fear of fear, explained the treatment process to the patients, and explained the meaning of the SUD and Validity of Cognition (VoC) scores which represent the level of disturbance of the memory, and the believability of the positive cognition used in EMDR therapy (Shapiro, 2017). Next, five treatment sessions were scheduled during which the following memories were processed: (i) antecedent or ancillary events that contributed to the phobia; (ii) the first time the fear was experienced; (iii) the most disturbing experience associated with the fear; (iv) the most recent time the fear was experienced. Also, any current triggers related to the physical sensations associated with the phobia were evaluated and integrated as a focus of treatment.

#### Waiting list control condition

After randomization, WLCC patients received a six-week reassessment appointment. If their symptoms worsened significantly, patients could contact a therapist *via* phone. Finally, after the intervention phase of this study, WLCC patients could be treated by psychologists in the university clinic.

### Assessment and outcome measures

#### Severity measure for acrophobia

The SMA is a self-report questionnaire developed to assess fear of high places (Azimisefat et al., 2021). Respondents are asked to indicate the extent of their agreement with each item on a Likert Grade on a five-choice scale ranging from 1 (“completely opposed”) to 5 (“completely agree”), resulting in a sum score between 14 and 70. This questionnaire has been administered and validated among 507 female students and 358 male students (Azimisefat et al., 2021). The results of the exploratory factor analysis showed that the SMA questionnaire consists of three subscales (cognitive, physical, behavioral and avoidance subscale). It includes five items for the cognitive subscale, four for the physical subscale, and five for the behavioral and avoidance subscale. Cronbach’s alpha coefficient of the SAM subscales obtained, respectively: 0/76, 0/79, and 0/80, and for the whole scale was 0/91 (Azimisefat et al., 2021). Test–retest reliability obtained on 50 people yielded an acceptable correlation coefficient (0.76).

#### Anxiety sensitivity index

The Anxiety Sensitivity Index (ASI-R) is a 36-item self-report scale developed by Taylor and Cox (1998) to assess fear of anxiety symptoms. Specifically, the scale is used to evaluate beliefs about the harmful consequences of anxiety symptoms and consists of six subscales (i.e., fear of cardiovascular symptoms, respiratory symptoms, gastrointestinal symptoms, publicly visible anxiety reactions, dissociative and neurological symptoms, and cognitive dyscontrol) (Taylor and Cox, 1998). Respondents are requested to indicate the extent of their agreement with each item on a Likert Grade on a five-choice scale, ranging from 0 (“very small”) to 4 (“very large”). The scores range between 0 and 144, indicating the lowest and highest scores, respectively. Cronbach’s alpha coefficient of the ASI-R in the current study was 0.91.

### Data analysis

After completing the interventions, data for 45 patients (EMDR group 15, VRET group 15, and WLCC group 15) were analyzed (response rate was 100%). SPSS was used to conduct all statistical analysis (IBM, version 23, Chicago IL, USA). Before analyzing the differences, we assured the homogeneity of the regression slopes, using Kolmogorov–Smirnov (K–S test) and Levine tests. All continuous variables were found to be normally distributed, according to the K–S test. The homogeneity assumption of the slopes of all variables were found to be insignificant. We may also assume that the state of equality of variances has not been observed since Levine’s test was not significant for all variables. We used parametric tests to analyze baseline differences between intervention groups. Analysis of covariance (ANCOVA) including pre-test symptoms of acrophobia and anxiety sensitivity scores as covariates was conducted to compare post-test symptoms of acrophobia and anxiety sensitivity scores between intervention groups. After the ANCOVA showed a significant overall group effect, the Tukey test was run to determine which specific groups’ means (compared with each other) were different.

## Results

### Demographics

Table 1 shows the demographic data regarding age, number of acrophobia situations, and severity of acrophobia symptoms in the study sample. A one-way ANOVA was used to identify age differences across the three conditions. No differences in age were found between VRET, EMDR, and WLCC groups.

### The efficacy of VRET and EMDR therapy on acrophobia symptoms and anxiety sensitivity

A comparison of the mean scores of the estimated post-test in the three groups based on analysis of covariance (ANCOVA) indicated a significant difference for at least one of the variables in severity of acrophobia and anxiety sensitivity in both the VRET group and EMDR therapy group. After controlling for covariates using ANCOVA, there were significant differences in overall acrophobia symptoms [F (2,41) = 14.415, *p* = 0.001] and anxiety sensitivity [F (2,41) = 32.681, p = 0.001] between experimental groups. Tukey’s range test showed a significant effect of experimental condition versus the WLCC. The effects of VRET and EMDR therapy on the acrophobia symptoms and anxiety sensitivity are shown in Table 3. The difference between pre-and post-treatment scores yielded a large effect for the reduction of symptoms of acrophobia (Cohen’s d = 1.08 for EMDR therapy and Cohen’s d = 1.03 for VRET) and anxiety sensitivity (Cohen’s d = 1.13 for EMDR therapy and Cohen’s d = 1.15 for VRET). Negligible differences were found between the two experimental groups after treatment (Cohen’s d = 0.13 for acrophobia symptoms and Cohen’s d = 0.03 for anxiety sensitivity).

**Table 3:** Means and standard deviations for the acrophobia symptoms and anxiety sensitivity at pre and post treatment by condition and between-condition comparisons.

Variable	Condition						Between subject group with ANCOVA								
	*EMDR (n = 15)*		*VRET (n = 15)*		*WLCC (n = 15)*		*EMDR* vs. *WLCC*			*VRET* vs. *WLCC*			*EMDR* vs. *VRET*		
	*Pre M (SD)*	*Post M (SD)*	*Pre M (SD)*	*Post M (SD)*	*Pre M (SD)*	*Post M (SD)*	*t*	*d*	*p*	*t*	*d*	*p*	*t*	*d*	*p*
Acrophobia symptoms	54.13 (3.87)	49.66 (4.30)	55.33 (5.81)	49.93 (5.88)	54.80 (4.16)	54.66 (3.88)	*−4.26*	*1.08*	*<0.001*	*−4.94*	*1.03*	*<0.001*	*0.68*	*0.13*	*0.77*
Anxiety sensitivity	86.33 (23.66)	60.26 (14.71)	81.00 (21.63)	57.06 (12.79)	82.46 (26.21)	*81.60 (25.87)*	*−7.05*	*1.13*	*<0.001*	*−6.93*	*1.15*	*<0.001*	*−0.12*	*0.03*	*0.99*

VRET = Virtual Reality Exposure Therapy; EMDR = Eye Movement Desensitization and Reprocessing therapy; WLCC = Waiting List control condition. The reported t, Cohen’s d and p concerns the differences between two groups via the Tukey test from the ANCOVA procedure. M: Mean; SD: standard deviation; t: T-statistic; d: Cohen’s *d* and p: *p*-value.

## Discussion

This trial is, to our knowledge, the first RCT demonstrating the efficacy of VRET and EMDR therapy for reducing symptoms of acrophobia and anxiety sensitivity. The results support our hypothesis as both VRET and EMDR therapy were found to be associated with significantly reduced symptoms of acrophobia and anxiety sensitivity in the experimental groups which was significantly greater than reductions found for the waiting list control condition. No significant differences were found regarding the reduction of acrophobia symptoms and anxiety sensitivity scores between the VRET and the EMDR therapy conditions most likely due to a lack of power to determine such differences.

Persons who completed VRET showed reduced acrophobia symptoms, which is in line with previous studies using this technology (Krijn et al., 2004; Boettcher et al., 2016; Celik et al., 2020; Krupić et al., 2020). In recent years, VRET has emerged as a viable alternative to *in vivo* exposure therapy as an evidence-based treatment for specific phobia, with the benefit of allowing patients to deal with fear in a controlled and safe environment. In the present study, patients were immersed in a virtual reality world with phobic scenarios, however, patients also knew that the situation was simulated, making it easier for them to face phobic situations. The therapist helped patients navigate the scenarios and provided education and support to develop new coping strategies to improve self-efficacy and to better tolerate situations that the patients had struggled with in real life. Perhaps most importantly, use of VRET simulations appeared to help patients recognize that no real threat or danger was present (Baus and Bouchard, 2014). In other words, repeated presentations of phobic stimuli (conditioned stimuli; CSs) without negative consequence (unconditioned stimulus; US) facilitated fear (conditioned response; CR) extinction, manifested behaviourally as reduced fear of heights. Also, from a more contemporary model of fear extinction one could argue that exposure was focused on patients’ feared outcomes, thereby maximizing the likelihood of that disaster expectation being violated and falsified (Craske et al., 2014).

EMDR therapy also proved effective in alleviating symptoms of acrophobia and anxiety sensitivity. This is in line with meta-analytic findings showing that EMDR has a positive effect on reducing the symptoms of fears and phobias (Yunitri et al., 2020). The theoretic foundation of EMDR therapy is ‘that current difficulties are caused by disturbing memories that are inadequately processed, and that symptoms are reduced or eliminated altogether when these memories are processed to resolution using dual attention bilateral stimulation’ (Laliotis et al., 2021). There is evidence to suggest that these forms of working memory taxation can enhance extinction through amygdala suppression (de Voogd and Phelps, 2020). As a result, disturbing memories can be reconsolidated to involve less emotional intensity which may explain why patients showed improvements in symptoms of acrophobia and anxiety sensitivity levels in the present study. To this end, the present findings are promising suggesting that a brief treatment of either VRET or EMDR therapy may be advantageous for those who suffer from acrophobia. Although the limited number of studies in this area provide no indication that EMDR therapy and VRET are inferior to standard cognitive behavioural therapy when it comes to the treatment of specific phobia, the results of the present study should not be overstated. Looking at the actual change in symptoms (SMA questionnaire), the EMDR therapy group yielded a reduction of 8.3% from baseline, whereas for the VRET group this was 9.8%. This means that both groups were only slightly below the inclusion criterion of 50 points after treatment. This suggests that both groups needed more treatment sessions to achieve not only a statistical effect, but also a better, clinically relevant effect.

The strengths of the present study include broad inclusion criteria, a study population (adolescents) that has so far been somewhat disregarded in the VRET and EMDR therapy literature, and applying EMDR to a phobia subtype that, as far as we know, until now has not been studied in a randomized controlled trial format. Conversely, the current study also has some limitations. Firstly, the present study used only self-report outcome measures and no behavioral testing in a real height situation for example, by using a behavioral approach task. Secondly, measurements indexing long term outcome effects were lacking and therefore unknown. Thirdly, the present study was conducted among female adolescents only, which calls for replication among male adolescents. Fourthly, the VRET that we used in this study contained four acrophobia scenarios, and all patients were exposed to those VR scenarios. Although this strategy helped maintain consistency and standardization of the intervention, the stimuli that trigger acrophobia symptoms may vary from person to person (Suyanto et al., 2017). As such, future studies should consider using a greater number and variety of scenarios that may better elicit acrophobic symptoms across participants. Lastly, although the data analyst and the person conducting the assessments were blind to the specific participant’s group, we could not blind therapists or patients to the interventions due to the observable differences between the two interventions.

In conclusion, the results of the present study suggest that both EMDR therapy and VRET are effective interventions for reducing symptoms of acrophobia and anxiety sensitivity in female adolescents when using a limited number of sessions. Further research evaluating the efficacy of EMDR therapy, and VRET for acrophobia is warranted. This should include not only ways to determine the long-term effects of the interventions, but also behavioural approach or avoidance tasks that examine to what extent participants are actually able to approach the feared situation after therapy (including measuring their heart rate, and observing their escape or avoidance strategies), thereby corroborating the information obtained by persons’ subjective fear ratings.

## Data availability statement

The original contributions presented in the study are included in the article/Supplementary material, further inquiries can be directed to the corresponding author.

## Ethics statement

The trial was approved by the Research Ethic Committees of Bushehr Province University of Medical Science (Reference: IR.BPUMS.REC.1400.036) and the Iranian Registry of Clinical Trials (IRCT20210213050343N1). Written informed consent to participate in this study was provided by the participants’ legal guardian/next of kin.

## Author contributions

PA conceptualized the study and performed the treatments. AJ cooperated in resource, editing, and review. SR supervised project and analyzed the data. PA, AJ, PK, and FJ drafted and/or revised the manuscript. SR contributed to the final approval of the manuscript. FJ contributed to the acquisition of data. All authors contributed to the article and approved the submitted version.

## Conflict of interest

AJ and SR receive income from published books on EMDR therapy and for training postdoctoral professionals in this method.

The remaining authors declare that the research was conducted in the absence of any commercial or financial relationships that could be construed as a potential conflict of interest.

## Publisher’s note

All claims expressed in this article are solely those of the authors and do not necessarily represent those of their affiliated organizations, or those of the publisher, the editors and the reviewers. Any product that may be evaluated in this article, or claim that may be made by its manufacturer, is not guaranteed or endorsed by the publisher.

## Footnotes

^1^ https://psycho-trauma.nl/wp-content/uploads/2015/08/EMDR-Therapy-for-specific-fears-and-phobias-The-phobia-protocol-De-Jongh2c-2015.pdf
