# Supplementary material for: Efficacy of virtual reality exposure therapy and eye movement desensitization and reprocessing therapy on symptoms of acrophobia and anxiety sensitivity in adolescent girls: A randomized controlled trial
Source: Front Psychol. 2022 Sep 15;13:919148. doi: 10.3389/fpsyg.2022.919148 (PMC9521642; doi:10.3389/fpsyg.2022.919148)
Supplement: Supplementary file 1 [file Data_Sheet_1.docx]

**TABLE 1. The VRET Protocol for Specific Phobias:**

**Training phase.** Through the head-mounted device (HMD), the patient viewed a 3-dimensional (3D), stereoscopic, simulated high place for five minutes, in which the patient moved her head, observed the surroundings, and walked in the environment.

**Experimental phase.** The standard protocols of cognitive behavioral therapy for specific phobias was conducted and the patient was exposed to four different VR scenarios (VR1 to VR4) in the following predetermined hierarchy:

*A) VR1.* The patient could walk on a high circular hill, observe the surroundings, and stand at the top of the peak to look down.

*B) VR2.* The patient was on a high roof and could walk on top of a building and go to the edge of the roof to observe the surroundings.

*C) VR3.* The patient was asked to climb a ladder and could gradually climb the ladder and look around.

*D) VR4.* The patient was riding a balloon that was gradually elevating from the ground and climbing in height. The patient could look down from the edge of the balloon. This scenario is based on a weakness in the acrophobic individual's ability to control her stability of posture given that persons with acrophobia are afraid of the sense of movement and height at the same time.

*E) Final* *VRET.* In the last VR exposure, the patient was placed in a combination of the various situations to make sure that all acrophobic scenarios were performed.

**Table 2. Demographic characteristics of the randomized controlled trial sample**

| Descriptive characteristics | EMDR group | VRET group | WLCC group | F | P |
| --- | --- | --- | --- | --- | --- |
| Age M (SD) | 16.93 (0.88) | 17.20 (0.77) | 16.93 (0.79) | 0.528 | 0.593 |
| Education attainment (N and %) |  |  |  |  |  |
| 10th Grade | 6 (40.0) | 3 (20.0) | 5 (33.33) |  |  |
| 11th Grade | 4 (26.66) | 6 (40.0) | 6 (40.0) |  |  |
| 12th Grade | 5 (33.33) | 6 (40.0) | 4 (26.66) |  |  |
| Severity of acrophobia symptoms (N and %) |  |  |  |  |  |
| Mild | 1 (6.66) | 0 (0.00) | 0 (0.00) |  |  |
| Average | 9 (60) | 9 (60) | 8 (53.33) |  |  |
| Severe | 5 (33.33) | 6 (40) | 7 (46.66) |  |  |
| Number of acrophobic situations (N and %) |  |  |  |  |  |
| Acrophobic situation #1 | 7 (46.66) | 2 (13.33) | 4 (26.66) |  |  |
| Acrophobic situations #2 | 1 (6.66) | 8 (53.33) | 4 (26.66) |  |  |
| Acrophobic situations #3 | 7 (46.66) | 5 (33.33) | 7 (46.66) |  |  |

***Note.*** M= mean, SD= standard deviation, N= number, VRET= Virtual Reality Exposure Therapy; EMDR= Eye Movement Desensitization and Reprocessing, WLCC = Waiting List Control Condition.

**Table 3. Means and standard deviations for the acrophobia symptoms and anxiety sensitivity at pre and post treatment by condition and between-condition comparisons**

| **Variable** | **Condition** | | | | | | **Between Subject Group with ANCOVA** | | | | | | | | |
| --- | --- | --- | --- | --- | --- | --- | --- | --- | --- | --- | --- | --- | --- | --- | --- |
|  | *EMDR (n=15)* | | *VRET (n=15)* | | *WLCC (n=15)* | | *EMDR vs. WLCC* | | | *VRET vs. WLCC* | | | *EMDR vs. VRET* | | |
|  | *Pre M (SD)* | *Post M (SD)* | *Pre M (SD)* | *Post M (SD)* | *Pre M (SD)* | *Post M (SD)* | *t* | *d* | *p* | *t* | *d* | *p* | *t* | *d* | *p* |
| **Acrophobia symptoms** | 54.13 (3.87) | 49.66 (4.30) | 55.33 (5.81) | 49.93 (5.88) | 54.80 (4.16) | 54.66 (3.88) | *-4.26* | *1.08* | *<.001* | *-4.94* | *1.03* | *<.001* | *0.68* | *0.13* | *0.77* |
| **Anxiety sensitivity** | 86.33 (23.66) | 60.26 (14.71) | 81.00 (21.63) | 57.06 (12.79) | 82.46 (26.21) | *81.60 (25.87)* | *-7.05* | *1.13* | *<.001* | *-6.93* | *1.15* | *<.001* | *-0.12* | *0.03* | *0.99* |

***Note.*** VRET= Virtual Reality Exposure Therapy; EMDR= Eye Movement Desensitization and Reprocessing therapy; WLCC= Waiting List control condition; M: Mean; SD: standard deviation; t: T-statistic; d: Cohen's d; p: P-value. The reported t, Cohen’s d and p concerns the differences between two groups via the Tukey test from the ANCOVA procedure.

**SPIRIT flow diagram**

|  | **STUDY PERIOD** | | |
| --- | --- | --- | --- |
|  | Enrollment | Post-Allocation | |
| TIME POINT |  | T0 | T1 |
| ENROLLMENT: |  |  |  |
| Screening  Informed consent  Allocation | X |  |  |
|  | X |  |  |
|  | X |  |  |
| INTERVENTIONS:  VRET Intervention  EMDR Intervention  Waitlist Control Condition |  |  |  |
|  |  |  |  |
|  |  |  |  |
|  |  |  |  |
| ASSESSMENTS:  SMA  ASI-R |  |  |  |
|  |  | X | X |
|  |  | X | X |

TIDieR (Template for Intervention Description and Replication) checklist

| **Item number** | **Item** | **Where located **** | |
| --- | --- | --- | --- |
|  |  | Primary paper  (page or appendix  number) | Other ^†^ (details) |
|  | **BRIEF NAME** |  |  |
| **1.** | Provide the name or a phrase that describes the intervention. | 10 | 12 |
|  | **WHY** |  |  |
| **2.** | Describe any rationale, theory, or goal of the elements essential to the intervention. | 6 | N/A |
|  | **WHAT** |  |  |
| **3.** | Materials: Describe any physical or informational materials used in the intervention, including those provided to participants or used in intervention delivery or in training of intervention providers. Provide information on where the materials can be accessed (e.g. online appendix, URL). | 10 | 12 |
| **4.** | Procedures: Describe each of the procedures, activities, and/or processes used in the intervention, including any enabling or support activities. | 10 | 11-12 |
|  | **WHO PROVIDED** |  |  |
| **5.** | For each category of intervention provider (e.g. psychologist, nursing assistant), describe their expertise, background and any specific training given. | 6 | N/A |
|  | **HOW** |  |  |
| **6.** | Describe the modes of delivery (e.g. face-to-face or by some other mechanism, such as internet or telephone) of the intervention and whether it was provided individually or in a group. | 9 | 6 |
|  | **WHERE** |  |  |
| **7.** | Describe the type(s) of location(s) where the intervention occurred, including any necessary infrastructure or relevant features. | 6 | N/A |
|  | **WHEN and HOW MUCH** |  |  |
| **8.** | Describe the number of times the intervention was delivered and over what period of time including the number of sessions, their schedule, and their duration, intensity or dose. | 6 | 12 |
|  | **TAILORING** |  |  |
| **9.** | If the intervention was planned to be personalised, titrated or adapted, then describe what, why, when, and how. | N/A | N/A |
|  | **MODIFICATIONS** |  |  |
| **10.^ǂ^** | If the intervention was modified during the course of the study, describe the changes (what, why, when, and how). | N/A | N/A |
|  | **HOW WELL** |  |  |
| **11.** | Planned: If intervention adherence or fidelity was assessed, describe how and by whom, and if any strategies were used to maintain or improve fidelity, describe them. | N/A | N/A |
| **12.^ǂ^** | Actual: If intervention adherence or fidelity was assessed, describe the extent to which the intervention was delivered as planned. | N/A | N/A |
